# Effects of the Caregiver Interaction Profile Training on Caregiver–Child Interactions in Dutch Child Care Centers: A Randomized Controlled Trial

**DOI:** 10.1007/s10566-016-9383-9

**Published:** 2016-11-30

**Authors:** Katrien O. W. Helmerhorst, J. Marianne A. Riksen-Walraven, Ruben G. Fukkink, Louis W. C. Tavecchio, Mirjam J. J. M. Gevers Deynoot-Schaub

**Affiliations:** 1 Research Institute of Child Development and Education, University of Amsterdam, P.O. Box 15780, 1001 NG Amsterdam, The Netherlands; 2 Behavioural Science Institute, Radboud University Nijmegen, P.O. Box 9104, 6500 HE Nijmegen, The Netherlands; 3 Kohnstamm Institute, P.O. Box 94208, 1090 GE Amsterdam, The Netherlands

**Keywords:** Child care center, Caregiver–child interactions, Caregiver training, Video feedback, Child care quality, Intervention study

## Abstract

**Background:**

Previous studies underscore the need to improve caregiver–child interactions in early child care centers.

**Objective:**

In this study we used a randomized controlled trial to examine whether a 5-week video feedback training can improve six key interactive skills of caregivers in early child care centers: Sensitive responsiveness, respect for autonomy, structuring and limit setting, verbal communication, developmental stimulation, and fostering positive peer interactions.

**Method:**

A total of 139 caregivers from 68 early child care groups for 0- to 4-year-old children in Dutch child care centers participated in this RCT, 69 in the intervention condition and 70 in the control condition. Caregiver interactive skills during everyday interactions with the children were rated from videotape using the Caregiver Interaction Profile (CIP) scales at pretest, posttest, and follow-up 3 months after the posttest.

**Results:**

Results at posttest indicate a significant positive training effect on all six caregiver interactive skills. Effect sizes of the CIP training range between *d* = 0.35 and *d* = 0.79. Three months after the posttest, caregivers in the intervention group still scored significantly higher on sensitive responsiveness, respect for autonomy, verbal communication, and fostering positive peer interactions than caregivers in the control group with effect sizes ranging between *d* = 0.47 and *d* = 0.70.

**Conclusions:**

This study shows that the quality of caregiver–child interactions can be improved for all six important caregiver skills, with a relatively short training program. Possible ways to further improve the training and to implement it in practice and education are discussed.

## Introduction

Numerous studies have demonstrated the importance of early child care quality for children’s socio-emotional and academic development (for an overview see Belsky et al. 2007; Vandell and Wolfe 2000; Vandell et al. 2010). High quality care can be characterized as care that contributes to children’s wellbeing and development (see e.g., Layzer and Goodson 2006). Caregivers play a pivotal role in determining the quality of young children’s everyday experiences in child care, both by their direct interactions with the children and by guiding the children’s interactions with peers and play materials. Given that many young children in the Western world attend formal child care nowadays (e.g., 56% of the 0- to 4-year-olds in the Netherlands; OECD 2014), it is important to improve the quality of caregiver–child interactions where needed. In the present study we examined whether six key caregiver skills in interacting with young children in child care centers can be improved using a 5-week onsite video feedback training for caregivers.

In the Netherlands, the quality of child care is periodically assessed with the internationally widely used Infant/Toddler Environment Rating Scale-Revised (ITERS-R; Harms et al. 2003) and Early Childhood Environment Rating Scale-Revised (ECERS-R; Harms et al. 1998). The ITERS-R and ECERS-R are useful in regular national quality assessments, because they provide a comprehensive picture of the quality of the child care environment in a broad set of domains, including caregiver–child interactions. In addition to this more global picture, we wanted to get a more in-depth picture of the quality of the interactions between caregivers and children, which is generally acknowledged as the core of child care quality (e.g., Vandell and Wolfe 2000). For that purpose, we needed an instrument that met the following requirements: (a) it should be relatively time efficient so that it could be completed together with the ITERS-R/ECERS-R during a single center visit; (b) it should assess individual caregivers’ skills in interacting with a *group* of children, thus taking into account caregivers’ ability to divide their attention and react consistently across children; (c) it should be applicable to interactions with children across the whole age range of 0–4 years, i.e., the predominant type of child care group in the Netherlands; (d) it should preferably be theory-based and supported by empirical evidence underscoring the included caregiver interactive skills to contribute to the well-being and development of 0- to 4-year-old children.

Based on a review of the relevant literature (reported in Helmerhorst et al. 2014) the following six caregiver interactive skills were selected to be included in the instrument. (1) *Sensitive responsiveness* refers to a caregiver’s ability to recognize children’s emotional and physical needs and respond appropriately and promptly to children’s cues and signals. This aspect of caregiver behavior is considered the key quality of caregiving in attachment theory (Ainsworth et al. 1978) and is generally recognized as the most basic aspect of caregiver behavior in interactions with children from birth onward. (2) *Respect for autonomy* is the extent to which a caregiver is nonintrusive and recognizes and respects the validity of children’s intentions and perspectives. Respecting a child’s autonomy becomes increasingly important as a caregiver skill during the second year of life, when acquiring a sense of autonomy is considered a central developmental issue in children’s development (Erikson 1950; Sroufe 1979). However, parental intrusiveness (i.e., lack of respect for autonomy) already in early infancy has been shown to independently contribute to the prediction of developmental problems at early school age (Egeland et al. 1993). (3) *Structuring and limit setting* refers to a caregiver’s ability to clearly communicate expectations toward children and set clear and consistent limits. This caregiver skill, which also becomes increasingly important in the second year of life as children quickly expand their locomotor abilities, contributes to the predictability of the environment and thereby to the development of feelings of security and competence (e.g., Thompson 1998). (4) *Verbal communication* refers to the frequency and quality of verbal caregiver–child interactions and has been shown to be important for children’s language, cognitive, and social development (e.g., Mashburn et al. 2008, NICHD ECCRN 2000, 2005). (5) *Developmental stimulation* refers to deliberate attempts of a caregiver to foster children’s development (e.g., motor skills, cognitive development, and creativity). Well-adjusted developmental stimulation in child care centers has been shown to foster children’s cognitive development already in the very first year of life (Albers et al. 2010). (6) *Fostering positive peer interactions* refers to the extent to which a caregiver facilitates, encourages, and stimulates positive interactions between children. Positive peer interactions in child care centers have been shown to contribute to children’s wellbeing and social development from infancy onwards (e.g., Gevers Deynoot-Schaub and Riksen-Walraven 2006; NICHD ECCRN 2001). For a more complete description of the selection and theoretical underpinning of the above six caregiver interactive skills see De Kruif et al. (2007) and Helmerhorst et al. (2014).

At that time (2005), there was no readily available measurement instrument to rate all six caregiver interactive skills at *group level* in child care groups with children across the whole age range of 0–4 years. Therefore, we composed a set of rating scales ourselves—the Caregiver Interaction Profile (CIP) scales—including adapted versions of four existing rating scales measuring comparable interactive skills, and two self-developed scales. The scales for rating sensitive responsiveness, respect for autonomy, and structure and limit setting were adapted from scales devised by De Schipper and Riksen-Walraven (2004) to rate caregiver behavior toward groups of 0- to 4-year-old children in child care centers (De Schipper et al. 2006). The scale for rating developmental stimulation was adapted from a scale included in the Observational Record of the Caregiving Environment (ORCE; see NICHD ECCRN 1996), and the scales for rating verbal communication and fostering positive peer interactions were self-developed.

For a more extensive description of the different steps in the development and validation of the CIP scales and how they are related to other comparable measurement instruments (such as the widely used CLASS, see Pianta et al. 2008a) we refer the reader to Helmerhorst et al. (2014).

After validation, the CIP scales were applied in a large nationally representative sample of caregivers in Dutch child care centers (De Kruif et al. 2009; Helmerhorst et al. 2015). The results showed caregivers to score highest, on average, on the three more “basic” caregiving skills, i.e., sensitive responsiveness, respect for autonomy, and structuring and limit setting, although about one third of the caregivers scored inadequate or moderate on these skills, indicating that there is still room for improvement. Scores were clearly lower for the more “educational” skills, i.e., verbal communication, developmental stimulation, and fostering positive peer interactions, with the majority of the caregivers scoring in the moderate to inadequate range. These results are in line with previous findings in the US (Guo et al. 2010; La Paro et al. 2009; Mashburn et al. 2008; Thomason and La Paro 2009), Australia (Tayler et al. 2013) and Spain (Sandstrom 2012). Results across all of these countries were highly similar: caregivers scored relatively low on instructional support compared to emotional support. This clearly underscores the need to improve caregivers’ skills by training and professional development (La Paro et al. 2009; LoCasale-Crouch et al. 2007; Mashburn et al. 2008).

Based on the above results we developed a training program for caregivers to improve the six interactive skills included in the Caregiver Interaction Profile scales. The present study examined the effects of this training using a randomized controlled trial.

### Previous Interventions to Improve Caregiver–Child Interactions

In designing the training program, we first reviewed earlier meta-analytic reviews of intervention studies to identify effective features of training programs for improving caregiver–child interactions (Fukkink and Lont 2007; Werner et al. 2016). The authors of both meta-analyses conclude that, on average, training significantly improved caregiver skills: Fukkink and Lont reported *d* = 0.40 for the aggregated effect on caregiver skills and Werner and colleagues found Hedges’ *g* = 0.35 (Hedges’ *g* is an effect size measure comparable to Cohen’s *d*, correcting for small sample bias; Hedges and Olkin 1985). However, not all training programs were effective; the meta-analysis of Fukkink and Lont (2007) also showed negative effect sizes for 28% of the studies.

The meta-analysis by Werner et al. (2016), which is the most recent of the two meta-analyses discussed here, reports on 16 RCT studies examining the effects of caregiver training to improve caregiver–child interactions. Studies were categorized by the focus of the intervention, using the same six caregiver interactive skills that are also included in the Caregiver Interactive Profile (CIP) training examined in the present study. Nine studies focused mainly on sensitive responsiveness and most of the described training programs also included some elements of respect for autonomy and structuring and limit setting. The remaining seven studies were mainly aimed at improving verbal communication and fostering positive peer interactions. The meta-analysis did not report any studies that targeted developmental stimulation nor any studies that targeted all six caregiver interactive skills. The overall effect size of the nine studies that were aimed at sensitive responsiveness was *g* = 0.54. The overall effect size for the seven studies that reported on training programs for verbal communication and fostering positive peer interactions was *g* = 0.30. Werner and colleagues conclude that the RCT-studies demonstrated that effect sizes were highest at the caregiver level (*g* = 0.44) and lower at classroom (*g* = 0.39) and child level (*g* = 0.26).

The meta-analysis of Fukkink and Lont (2007) focused on 17 studies between 1980 and 2005 and included only two relatively recent RCT-studies (Girolametto et al. 2003, 2004) that were not included in the earlier discussed meta-analysis by Werner et al. (2016). Both RCT studies examined a training that used individualized video feedback, and showed significant effects of training on caregivers’ interactions with children. Trained caregivers were more verbally supportive and better able to facilitate peer interactions (Girolametto et al. 2003, 2004) as compared to the caregivers in the control groups that received a training without video feedback (Girolametto et al. 2004) or a training with video feedback but with a different content (Girolametto et al. 2003). Effect sizes ranged between *d* = 0.30 and *d* = 0.50 for caregiver’s verbal promoting techniques.

Both meta-analyses also identified some characteristics of the training program that are related to more favorable outcomes. First, Werner et al. (2016) found that individual caregiver training programs were more effective (*g* = 0.41) than programs without individual training (*g* = 0.09). Furthermore, programs with more than 10 training hours in total did not differ in their effectiveness from programs with less than 10 h of training in total. Werner et al. (2016) did not specifically investigate the effectiveness of individual video feedback, but inspection of the effect sizes of studies with individual video feedback programs showed that these were all above the average effect size across all studies in the meta-analysis. Fukkink and Lont (2007) found the largest effects for programs with a fixed curriculum (i.e. identical content of the training across trainees) and programs including fewer trainees. Large-scale programs designed for a variety of training formats and for a wide variety of learners were less effective, possibly because this negatively affects implementation fidelity (Fukkink and Lont 2007).

In our further search for RCT studies examining the effects of caregiver training programs to promote caregiver–child interactions, we found two studies that were not included in the abovementioned meta-analyses. First, Pianta et al. (2008b) evaluated the web-mediated MyTeachingPartner program (MTP), which includes video exemplars and individualized feedback to videotapes made by the caregivers themselves. The consultant edited the tapes in short 1–2 min segments and provided these segments with written feedback that focused on caregivers’ interactive skills with the children. Furthermore, the consultant asked the caregivers questions to call specific attention to their behaviors. Caregivers answered these questions online. Every other week, caregivers and consultants video-chatted online to discuss the feedback. The MTP program is based on the conceptual framework of the CLASS (Pianta et al. 2008a) for defining classroom interactions. Pianta et al. (2008b) demonstrated that teachers who received the individualized MTP consultation for an entire year showed significantly greater increases in the quality of their interactions than teachers in the web-only control condition who only had access to video clip examples of high-quality interactions. The authors did not provide effect sizes, but they reported in the discussion of the paper that effect sizes were small. The second RCT study was conducted by Piasta et al. (2012), who evaluated the effects of a personal development program for preschool teachers to improve their conversational responsivity. The program included workshops and, every 2 weeks, written individual feedback on the teachers’ own videotaped classroom interactions. Piasta et al. (2012) demonstrated that the teachers who received the program showed significantly larger improvements in communication-facilitating strategies than teachers in a comparison group who received a comparable program that was not specifically focused on their conversational responsivity (*d* = 0.61).

Altogether, results from the two meta-analyses and the two additional intervention studies with an RCT design show that it is possible to improve caregivers’ interactive skills through training. In general, effect sizes in these studies are moderate. Although the studies are difficult to compare, their results allow two main conclusions regarding the intervention that we aimed to develop.

First, with regard to the intervention method, individual video feedback on caregivers’ own everyday interactions with the children in their group appears to be an effective method (Domitrovich et al. 2009; Fukkink and Lont 2007; Fukkink and Tavecchio 2010; Pianta et al. 2008b; Piasta et al. 2012; Werner et al. 2016). Previous meta-analyses also indicated that video feedback as a core intervention method is more effective at improving parents’ interactions skills than training programs without video feedback (Bakermans-Kranenburg et al. 2003; Fukkink 2008) and professionals’ interactions skills (Fukkink et al. 2011). We therefore decided to adopt this method.

Second, with regard to the duration of the training, the programs reviewed above showed a broad variation in duration, from four sessions to an entire year. The intervention studies focusing on professional caregivers demonstrated that relatively short programs (less than 10 h in total) may be as effective as longer lasting programs (more than 10 h in total; see Werner et al. 2016). This is in line with the conclusion by Bakermans-Kranenburg et al. (2003) from a meta-analysis of intervention programs aimed at improving parent–child interactions. They suggest that interventions with fewer than 5 sessions (*d* = 0.42) can be at least as effective as interventions with a larger number of sessions (between 5 and 16 sessions; *d* = 0.38). In accordance with this, we opted for a relatively short training with 5 weekly 2-h (10 h in total) sessions.

### The Present Study

In sum, based on earlier studies with the CIP scales in Dutch child care centers and on the results of previous studies examining the effects of caregiver and teacher training, we developed a 5-week video feedback training for caregivers, based on the conceptual framework underlying the CIP scales and aiming to improve the six CIP skills. The present RCT study examined whether the CIP training program was effective in improving the six caregiver interactive skills. This study is different from previous studies on the effectiveness of training programs to improve caregiver–child interactions in at least two ways. First, this is the first study to examine the effects of a training program focusing on the entire present set of six caregiver skills that, based on developmental theory and research, are generally seen as important for young children’s wellbeing and development (see Helmerhorst et al. 2014; Werner et al. 2016). Previous training programs have focused only on a selection of the six caregiver interactive skills, but none was aimed at improving all six skills. Second, this study examined the effects of a training that aims to improve caregiver skills in interaction with children in the whole age range between 0 and 4 years. The above review of effect studies shows that most of the earlier studies pertained to preschool teachers of 2- to 5-year-old children. As a result, there is only little research evidence for caregivers of children in the entire age range of 0–4 years; the present study aims to fill this gap by focusing on caregiver interactive skills that are relevant for children across the whole 0–4 years age range Finally, the current study also presents follow-up results of the CIP training program. The vast majority of the abovementioned RCT studies reported only posttest results, directly after the training program. The present study therefore sheds more light on long-term effects of training programs to promote the quality of caregiver–child interactions.

## Method

### Participants and Randomization

Child care groups (i.e., groups of children who attend child care together in the same classroom) in this study were recruited between September 2010 and September 2012 from child care centers in and around the city of Amsterdam, the Netherlands. Directors of the child care centers responded to appeals in (digital) newsletters and announcements on child care websites, which also included the eligibility criteria: (1) child care centers had to have mixed-age groups and (2) had to participate with an even number of groups to assign to the study. In the Netherlands, three types of child care groups are distinguished: infant groups (0- to 2-year-olds), preschool groups (2- to 4-year-olds), and mixed-age groups (0- to 4-year-olds). We focused on mixed-age groups because they allowed to test the effects of the intervention with children across the whole possible age range, and because mixed-age groups are most prevalent in the Netherlands (see Helmerhorst et al. 2015).

**Fig. 1:**
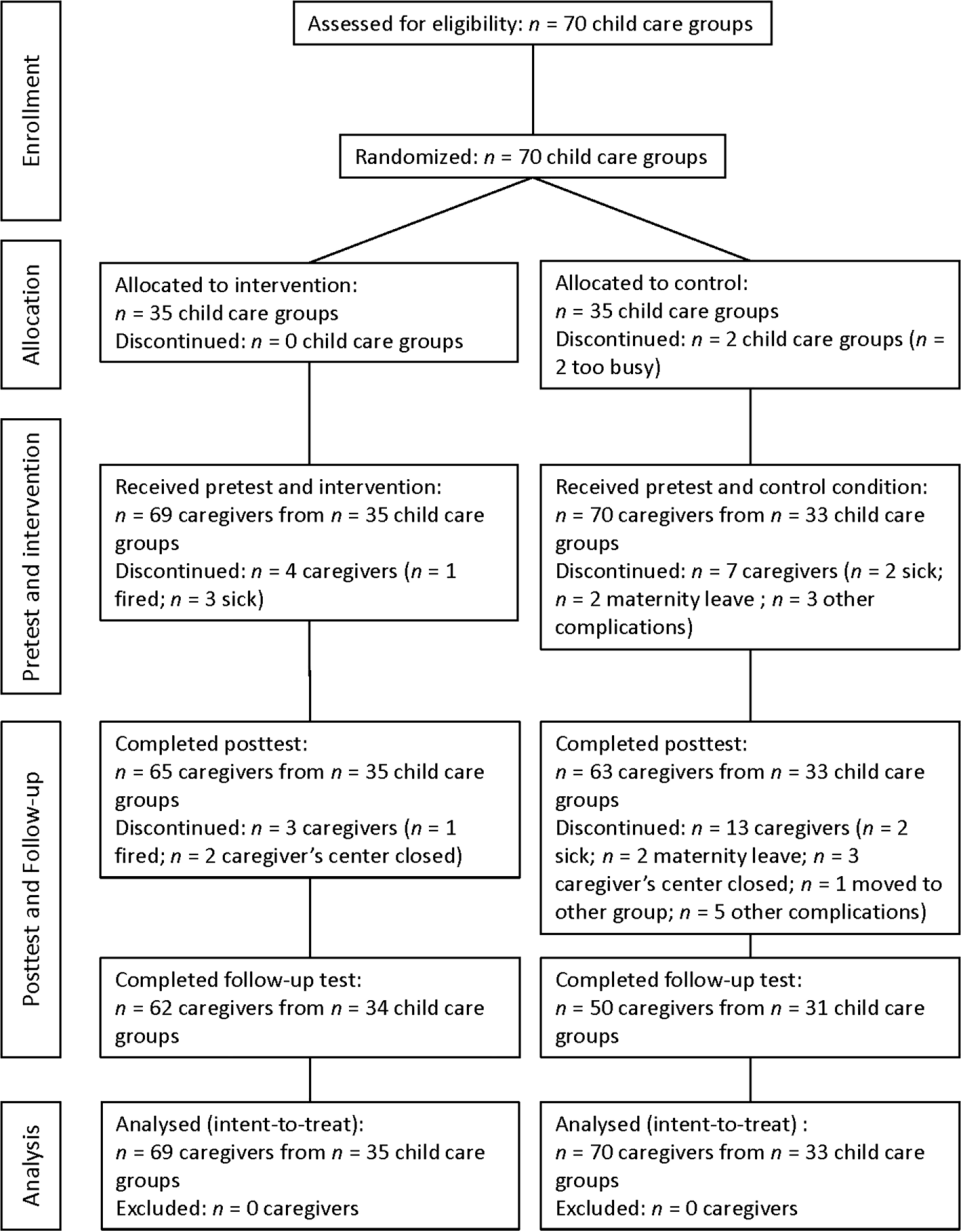
Flow chart of participants (child care groups and caregivers) through the study

The original sample (see flow chart, Fig. 1) included 70 child care groups, half of which were randomly allocated to the intervention condition and half to the control condition (two-arm parallel trial design, blocked randomization with allocation ratio 1:1). Randomization was performed by the first author. Randomization was initially done at the level of the care group; center, directors applied with an even number of care groups of which half were randomly assigned to the intervention condition and half to the control condition. Halfway through the study, applications to participate in the study came mostly from child care organizations applying with multiple centers. In these cases, randomization was done at center level: per child care organization, half of the centers (with all participating care groups) were randomly assigned to the intervention condition and half to the control condition. After randomization and before the pretest, two care groups assigned to the control condition dropped out (‘too busy’). This brought the final sample to 68 child care groups from 33 centers, with 35 groups in the intervention condition and 33 in the control condition.

All caregivers in these 68 care groups were invited to participate using an informed consent procedure. Two caregivers—both from care groups in the control condition—did not want to be filmed and were therefore not included in the study. Informed consent was obtained from all individual participants included in the study. The parents of the children in the selected care groups were also asked to give their informed consent for the filming procedures. Whenever parents did not give permission to film their children, we made sure that these children were not filmed. As a result, care groups in which not all parents gave their consent did not have to be excluded from the study.

The final study sample included 139 caregivers, 69 in the experimental condition and 70 in the control condition. In 57 child care groups two caregivers participated, in 7 groups (all in the control condition) three caregivers, and in 4 groups (1 in the experimental condition and 3 in the control condition) only one caregiver participated. In the Netherlands, there is no typical allocation of function (lead or assistant structure) between caregivers in the same care group. Caregivers were all female (just as in a nationally representative sample of caregivers drawn in a previous Dutch child care study; De Kruif et al. 2009; Helmerhorst et al. 2015) and were on average 32.4 years old (*SD* = 9.65, range 18–56), worked 28.3 h a week (*SD* = 6.44, range 16–40), and had 8.2 years (*SD* = 6.48, range 0–25) of working experience in child care. The majority of the caregivers (89.5%) had completed the regular 3-year vocational training in general social-pedagogic work, 7% had a bachelor degree, and 3.5% worked in practice as part of their education. There were no significant differences between caregivers in the experimental and control condition at pretest for caregivers’ age, education, years of experience, and working hours per week. Furthermore, comparison of demographic characteristics of caregivers in the present study and with those of caregivers in a large nationally representative sample of caregivers (Fukkink et al. 2013) revealed no significant differences. The average group size at pretest was 10.7 children per group (*SD* = 2.17), with an average child–caregiver ratio of 4.8 children per caregiver (*SD* = 0.83). The average child–caregiver ratio in the experimental condition (*M* = 5.0, *SD* = 0.78) was significantly higher than in the control condition (*M* = 4.7, *SD* = 0.87), *t* = 2.14, *p* < .05, indicating that the ratios were more favorable in the control condition.

Figure 1 shows the flow chart of the participants (in terms of care groups and caregivers) through the study. As can be seen, a total of 27 (19%) of the 139 participating caregivers dropped out between pretest and follow-up test, 7 in the experimental condition and 20 in the control condition. Main reasons for dropping out were maternity leave, caregivers being sick at the day of the observation, and a child care center closing (one of the 33 centers closed between posttest and follow-up test). To check whether caregivers who dropped out during the study differed from those who stayed engaged, we conducted a MANOVA with dropout versus non-dropout and condition (experimental versus control) as between-subjects factors and pretest scores on all six CIP scales as dependent variables. Dropouts did not differ from non-dropouts on the pretest scores of the CIP scales. There was also no significant interaction effect between condition and dropout, indicating that there was no differential attrition in the experimental versus control condition.

### Design and General Procedure

This study is an RCT with random assignment of the child care groups to the experimental or control condition. Caregivers’ performance on the six CIP scales was the dependent variable in this study. Their performance was rated at pretest, at a posttest immediately after the intervention, and at a follow-up test three months after the posttest.

Parallel with the present training for caregivers to improve the quality of caregiver–child interactions, a separate consultancy program for center directors (i.e., managers of the child care center and responsible for finances, planning, and pedagogical policy and quality trough supervision of the caregivers) was conducted for the same care groups in which the caregivers received the CIP training. This consultancy program, comprising three consultations in total, was thus not provided to the caregivers but only to the center directors. The consultancy program also had a different aim than the CIP training for caregivers, i.e., improving the global quality of the child care environment as measured with the ITERS-R/ECERS-R (such as space and furnishings, play materials, and program) (see Helmerhorst et al. 2016). When examining the effects of the training for caregivers on their interactive skills as measured with the CIP scales in this study, we controlled for possible effects of the consultancy program (see “ITERS-R/ECERS-R” section).

For the pretest, posttest, and follow-up assessments, a trained experimenter visited the groups from 8 A.M. until approximately 3 P.M. between January 2010 and November 2013. During the visit, each individual caregiver was filmed for 8–10 min for later observation of their interactive skills in four different situations: diapering, lunch/snack, structured play, and transition between group activities. We decided to film different situations during the day to capture caregiver interactive skills in different parts of the daily child care program and to provide a more robust picture of a caregiver’s behavior during the day. The video episodes were rated afterwards by observers who had not visited the care group in question. Further, a trained experimenter utilized the ITERS-R and ECERS-R during the visit to the care group to assess the global quality of the child care environment. All observers and experimenters were extensively trained bachelor and master students from our university and were blind to the allocation of the care groups.

Two weeks after the pretest, the 5-week training program started at the same day of the week as the pretest. One week after the last training session or 6 weeks after the pretest for the control groups, each group was visited for the posttest by a second experimenter. Three months after the posttest each group was visited by a third experimenter for the follow-up test. For an optimal comparison, the posttest and follow-up visits were planned on the same day of the week as the pretest. At the pretest, caregivers also completed a questionnaire to collect individual background information (e.g., age, education, work experience). The authors declare that they have no conflict of interest. The study, involving caregivers and children of the care group, was approved by the Ethics Committee of the Faculty of Social and Behavioral Sciences of the University of Amsterdam.

### The Intervention

#### Experimental Group

The Caregiver Interaction Profile (CIP) training was developed by the Netherlands Consortium for Child Care Research, based on the conceptual framework underlying the CIP scales (for details on the framework see De Kruif et al. 2007; Helmerhorst et al. 2014). The CIP scales rate six caregiver skills in interacting with 0- to 4-year-old children in a group setting: *sensitive responsiveness*, *respect for autonomy*, *structuring and limit setting*, *verbal communication, developmental stimulation,* and *fostering positive peer interactions* (see “Method” section). Five CIP trainers (*n* = 5) with a bachelor (*n* = 2) or master degree (*n* = 3) in psychology trained the caregivers at the child care centers during work time. All trainers had been trained by the first author. The training for trainers consisted of five sessions in total, including video examples, home assignments and a training manual which described the standard set-up per session (see below) in detail.

Table 1 provides an overview of the CIP training. All CIP trainers used a manual with a standardized protocol for delivering the training. The training comprises five onsite visits in total, each lasting 2 h per caregiver. The first four visits were individual training sessions with the caregiver receiving feedback on her own videotaped interactions. One caregiver was trained during the morning and the second caregiver in the afternoon. The fifth and final training session was provided to both caregivers of the care group together; the session was cancelled if only one caregiver per group participated in the training. Before each training session, the trainer had analyzed video recordings to be used during that session. For the first session, the trainer used the pretest recording, and for the second, third and fourth session the trainer collected new video material of the caregiver during daily routines in the care group, after the training session. As can be seen in Table 1, the first session was used to inform the caregiver about the upcoming training sessions and procedures, which were described in an intervention booklet that all caregivers received at the start of the first session. Directly after this general introduction, the skills *sensitive responsiveness* and *respect for autonomy* were discussed, because those are considered to be the most basic aspects of caregiver behavior in interactions with children.

**Table 1:** Overview of sessions and content of the CIP training

Session	Content	Individual/with colleague	Video episodes collected
1	Information about procedures and general introduction Video feedback *sensitive responsiveness and respect for autonomy* Setup video feedback Read description CIP skill Caregiver rates video examples model in high, medium, low Watch caregiver’s own video episodes Caregiver fills in behavior checklist	Individual	Pretest
2	Review *sensitive responsiveness and respect for autonomy* Video feedback *structuring and limit setting and verbal communication* Setup video feedback: see session 1	Individual	After session 1
3	Review *structuring and limit setting and verbal communication* Video feedback *developmental stimulation and fostering positive peer interactions* Setup video feedback: see session 1	Individual	After session 2
4	Review *developmental stimulation and fostering positive peer interactions* Video feedback booster—two skills of caregiver’s own choice Setup video feedback: see session 1	Individual	After session 3
5	Learn from each other—caregivers choose three to five of their own video episodes to show their fellow-caregiver	With colleague	Pretest; after session 1, 2, 3

The set-up of the first four sessions was structured in a similar format: (1) first the trainer and caregiver read a description and discussed the relevant CIP skill, followed by (2) three video case examples (one high, one moderate and one low example) of a model caregiver interacting with children. The caregiver was requested to rate the video examples as high, medium, or low in terms of the relevant CIP scale. A description for high, medium and low performance was given in the intervention booklet. After the caregiver had rated the examples, (3) the trainer showed the caregiver short fragments (between 1 and 3 min) of the earlier selected video recordings of the caregiver’s own interactions. After watching the video fragment together, the trainer asked the caregiver to comment on her own video. Based on the caregiver’s reaction, the trainer and caregiver discussed the caregiver’s behavior, and when needed reviewed the episode again. Next, (4) the caregiver was asked to indicate three goals for the upcoming week using a checklist (also in the booklet), which listed concrete behaviors related to the specific CIP skill (e.g., make eye contact with the children, use a warm and calm voice when talking to the children for sensitive responsiveness).

The second training session started with a short review and follow-up on the goals as indicated by the caregivers during the previous training for sensitive responsiveness and respect for autonomy, followed by the same training for two new behaviors: *structuring and limit setting* and *verbal communication*. The third session again started with a review and follow-up on the goals for structuring and limit setting and verbal communication, and proceeded with *developmental stimulation* and *fostering positive peer interactions*. After the third session, the caregiver determined which two out of the six CIP skills she wanted to repeat during the fourth session. The vast majority of caregivers chose to repeat developmental stimulation and fostering positive peer interactions. Whenever a caregiver did not choose to repeat developmental stimulation and fostering positive peer interactions, these skills were nevertheless repeated during the start of the fourth session to ensure that all six skills were discussed two times with all caregivers during the intervention. After the fourth session, the caregiver was asked to choose three to five video episodes that she wanted to show to her colleague during the last session. The fifth and final session was used to share experiences and video episodes between both caregivers. This way, caregivers learned from each other by seeing what the fellow-caregiver had been working on during the past 4 weeks.

#### Control Group

Caregivers in the control group received no training at all and were only contacted for pretest, posttest, and follow-up test observations.

### Measures

#### Caregiver Interaction Profile (CIP) Scales

The CIP scales measure six caregiver interactive skills: (1) *sensitive responsiveness* refers to the extent to which a caregiver recognizes children’s individual emotional and physical needs, and responds appropriately and promptly to their cues and signals; (2) *respect for autonomy* refers to the extent to which a caregiver is non-intrusive but instead recognizes and respects the validity of children’s intentions and perspectives; (3) *structuring and limit setting* refers to the ability of a caregiver to clearly communicate expectations towards children and structure the situation accordingly and to set clear and consistent limits to the children’s behavior; (4) *verbal communication* refers to the frequency and quality of verbal interactions between caregiver and children; (5) *developmental stimulation* concerns the degree to which a caregiver deliberately attempts to foster children’s development, e.g., motor skills, cognitive development, and creativity; (6) *fostering positive peer interactions* refers to the extent to which the caregiver guides or facilitates positive interactions between children in the child care group. Each of the six CIP skills is rated on a single 7-point Likert scale, indicating the extent to which a caregiver demonstrates the skill (7 = *very high*, 6 = *high*, 5 = *moderate/high*, 4 = *moderate*, 3 = *moderate/low*, 2 = *low*, 1 = *very low*), with detailed behavioral descriptions for each of the seven scale points. In line with the behavioral descriptions of the scale points, scores of 5 and beyond are considered as “adequate to good”, and scores of 3 and below are considered as “inadequate”. The CIP scales have been shown to be reliable and valid for use in child care centers for 0- to 4-year-old children (see De Kruif et al. 2007; Helmerhorst et al. 2014).

Thirteen trained observers independently rated the behavior of the caregiver on the six 7-point scales for each of the four videotaped episodes. Observers who rated the video episodes had not visited the care group for data collection and were blind to the assignment of the condition. Per caregiver a mean score for each of the six skills was calculated by averaging across the four episodes (correlations between the caregiver mean scores in the four situations at pretest were all significant and ranged between 0.29 for lunch/snack and structured play and 0.48 for lunch/snack and transition). Observer training on the CIP scales comprised six 4-h sessions: per session two scales were discussed by means of example videos. In addition, observers had to rate a total of 36 videos (lasting 10 min each) in total and had to meet an 80% agreement within one scale point with a consensus score provided by experts. After initial training, inter-rater reliability (i.e., intraclass correlations computed for 10% of the tapes) was 0.87 on average (range 0.71–1.0). Correlations between the six CIP-scales at pretest were all significant and moderate, ranging between *r* = 0.23 and *r* = 0.67.

#### ITERS-R/ECERS-R

Both the Infant/Toddler Environment Rating Scale-Revised (ITERS-R; Harms et al. 2003, for children younger than 30 months) and the Early Childhood Environment Rating Scale-Revised (ECERS-R; Harms et al. 1998, for children between the ages of 30 and 48 months) were used in each child care group to assess global child care quality. This was done to control for possible effects of the parallel consultancy program for center directors, that was aimed at increasing global quality of child care as measured with these instruments. We applied only those ECERS-R/ITERS-R subscales and items that were addressed during the consultancy: Space and furnishings, Language, Activities, Interactions and Program structure. Subscale items are rated on a 7-point scale with descriptors for the scores 1 (inadequate), 3 (minimal), 5 (good), and 7 (excellent). Total scores for the ITERS-R (26 items) and for the ECERS-R (31 items) at pretest, posttest, and follow-up were computed by averaging item scores across subscales. Given their high correlation (*r* = 0.87), we aggregated the ITERS-R and ECERS-R total scores. Per care group, two ITERS-R/ECERS-R gain scores were subsequently computed; first, by subtracting the group’s combined total ITERS-R/ECERS-R score on the pretest from the total score on the posttest and second, by subtracting the group’s combined total ITERS-R/ECERS-R score on the pretest from the total score on the follow-up test. These gain scores were included as a control variable in our analyses testing the effects of the CIP training in the present study.

### Data Analysis

We estimated, a priori, that a sample of 140 caregivers (*N*_exp_ = *N*_con_ = 70) was adequate to test the effect of our intervention with a one-sided test at the convenient level of α = 0.05 and a statistical power of β = 0.80, assuming an experimental effect of *d* = 0.40; this experimental effect was based on the meta-analytic effect of 0.40 for skills from the meta-analysis of Fukkink and Lont (2007). Power was still estimated to be adequate, taking into account intracorrelation (*ICC* = −0.10 − 0.20).

Because caregivers (level 1) were nested within groups (level 2), and groups were nested within centers (level 3), we conducted multilevel analysis. Continuous data were standardized, so that the parameter estimate of the dichotomous variable (experimental versus control condition) can be interpreted as an effect size (Cohen’s *d*) while controlling for other parameter estimates in the analysis.

We examined the effects of the CIP training using an *intent*-*to*-*treat* analysis, in which all data for all caregivers were analyzed, regardless of attrition. As can be seen in Fig. 1, a total of 7 caregivers (10%) in the experimental group discontinued between pretest and follow-up (4 caregivers between pretest and posttest and 3 between posttest and follow-up). In the control group a total of 20 caregivers (29%) discontinued (7 caregivers between pretest and posttest and 13 between posttest and follow-up). Analysis using Little’s MCAR test demonstrated that the data were missing completely at random (*p* = .27 for the experimental group and *p* = .34 for the control group). Subsequently, we used Expectation Maximization to estimate missing parameters for the experimental group and control group separately (Schafer and Graham 2002). Finally, we analyzed the data files with both the imputed data and the original collected data and found no differences between the two analyses.

## Results

Table 2 shows the means and standard deviations for the six CIP scales in the experimental and control group at pretest, posttest, and follow-up. At pretest caregivers’ mean scores were moderate to high for sensitive responsiveness, respect for autonomy, and structuring and limit setting. The mean score for verbal communication was moderate, while caregivers scored low, on average, on developmental stimulation and fostering peer interactions. Caregivers scored lowest on fostering positive peer interactions.

**Table 2:** Mean scores and standard deviations for the experimental and control group at pretest, posttest, and follow-up test

	Experimental group		Control group	
	*N*	*M* (*SD*)	*N*	*M* (*SD*)
*Pretest*				
Sensitive responsiveness	69	5.04 (0.86)	70	5.00 (0.64)
Respect for autonomy	69	4.58 (0.77)	70	4.69 (0.77)
Structuring and limit setting	69	5.09 (0.82)	70	4.91 (0.85)
Verbal communication	69	3.94 (0.76)	70	3.80 (0.62)
Developmental stimulation	69	2.61 (0.82)	70	2.43 (0.75)
Fostering peer interactions	69	2.17 (0.85)	70	1.96 (0.77)
*Posttest*				
Sensitive responsiveness	65	5.33 (0.66)	63	4.78 (0.78)
Respect for autonomy	65	4.92 (0.85)	63	4.48 (0.73)
Structuring and limit setting	65	5.24 (0.84)	63	4.98 (0.92)
Verbal communication	65	4.25 (0.86)	63	3.72 (0.73)
Developmental stimulation	65	3.02 (0.94)	63	2.25 (0.65)
Fostering peer interactions	65	2.63 (1.06)	63	1.79 (0.76)
*Follow*-*up*				
Sensitive responsiveness	62	5.27 (0.80)	50	4.82 (0.82)
Respect for autonomy	62	4.91 (0.77)	50	4.39 (0.76)
Structuring and limit setting	62	5.16 (0.81)	50	4.93 (0.86)
Verbal communication	62	4.03 (0.76)	50	3.46 (0.69)
Developmental stimulation	62	2.53 (0.82)	50	2.33 (0.77)
Fostering peer interactions	62	2.22 (0.85)	50	1.80 (0.71)

### Effects of the CIP Training at Posttest

First, to examine the effects of the training at posttest for each of the six scales, we conducted multilevel analysis using MLwiN to test whether the CIP scores at posttest differed between the experimental and control group, controlling for the mean pretest CIP scores and controlling for the ITERS-R/ECERS-R gain score (to control for possible effects of the consultancy training for child care directors). The dependent variable in each model was the posttest score for the six CIP scales. Three variables were entered as predictors: a group (dummy) variable which indicated whether the group was allocated to the experimental or the control condition (0 = control, 1 = experimental), the mean pretest score of the CIP skill, and the ITERS-R/ECERS-R pretest–posttest gain score to control for possible effects of the parallel consultancy intervention.

Table 3 shows the outcomes of the six multilevel analyses for the separate CIP skills at posttest. As shown in the table, there was a significant difference between the experimental and control group at posttest for all of the CIP skills, indicating that the training had a positive effect on sensitive responsiveness, respect for autonomy, structuring and limit setting, developmental stimulation, and fostering positive peer interactions. Effect sizes (see CIP training estimates in Table 3) ranged between 0.35 for structuring and limit setting and 0.79 for fostering positive peer interactions. According to Cohen (1988) (*d* = 0.20 is a small effect, *d* = 0.50 is a medium effect, and *d* = 0.80 is a large effect), effects for sensitive responsiveness, respect for autonomy, verbal communication, developmental stimulation, and fostering positive peer interactions at posttest were medium to large and the effect for structuring and limit setting was small to medium.

**Table 3:** Effects of CIP training on caregiver interactive skills at posttest (multilevel analysis, N = 139)

	Sensitive responsiveness model		Respect for autonomy model		Structuring and limit setting model		Verbal communication model		Developmental stimulation model		Fostering peer interactions model	
	Estimate	*SE*	Estimate	*SE*	Estimate	*SE*	Estimate	*SE*	Estimate	*SE*	Estimate	*SE*
*Fixed parameters*												
Intercept	−0.35*	0.12	−0.37*	0.12	−0.16	0.14	−0.30*	0.14	−0.32*	0.13	−0.39*	0.13
CIP training	0.72*	0.17	0.67*	0.16	0.35*	0.18	0.59*	0.19	0.74*	0.15	0.79*	0.18
ITERS-R/ECERS-R gain score	0.03	0.09	−0.06	0.09	−0.01	0.10	0.03	0.10	0.06	0.09	−0.03	0.09
SR pretest	0.27*	0.07	–		–		–		–		–	
RA pretest	–		0.22*	0.08	–		–		–		–	
SL pretest	–		–		0.10	0.08	–		–		–	
VC pretest	–		–		–		0.23*	0.07	–		–	
DS pretest	–		–		–		–		0.12	0.07	–	
FPPI pretest	–		–		–		–		–		0.20*	0.07
*Random parameters*												
Center level	0.07	0.11	0.10	0.11	0.20	0.15	0.00	0.00	0.31	0.11	0.00	0.00
Group level	0.16	0.13	0.01	0.13	0.12	0.15	0.43	0.12	0.00	0.00	0.34	0.10
Caregiver level	0.56	0.09	0.73	0.12	0.64	0.11	0.46	0.08	0.52	0.08	0.44	0.07
*Deviance*	353.81		360.76		377.39		357.86		350.72		344.12	

*SR* sensitive responsiveness, *RA* respect for autonomy, *SL* structuring and limit setting, *VC* verbal communication, *DS* developmental stimulation, *FPPI* fostering positive peer interactions

* *p* < .05

### Effects of the CIP Training at Follow-up

Next, we analyzed whether the effects of the training were retained at follow-up, three months after completion of the training. Multilevel models for the analysis of the follow-up results were comparable to the posttest models, with the follow-up scores on the CIP scales as outcome measure. Table 4 shows the outcomes of the multilevel analyses for each CIP skill. The results show that there was still a significant difference between the experimental and control group at follow-up for four of the six scales: sensitive responsiveness, respect for autonomy, verbal communication, and fostering positive peer interactions. For structuring and limit setting and developmental stimulation, the difference between experimental and control groups was no longer significant at follow-up. Effect sizes at follow-up (see CIP training estimates in Table 4) were medium to large for respect for autonomy, verbal communication, and fostering positive peer interactions, and medium for sensitive responsiveness.

**Table 4:** Effects of CIP training on caregiver interactive skills at follow-up (multilevel analysis, N = 139)

	Sensitive responsiveness model		Respect for autonomy model		Structuring and limit setting model		Verbal communication model		Developmental stimulation model		Fostering peer interactions model	
	Estimate	*SE*	Estimate	*SE*	Estimate	*SE*	Estimate	*SE*	Estimate	*SE*	Estimate	*SE*
*Fixed parameters*												
Intercept	−0.25*	0.12	−0.35*	0.11	−0.11	0.13	−0.34*	0.12	−0.15	0.14	−0.33*	0.12
CIP training	0.47*	0.17	0.70*	0.16	0.22	0.18	0.68*	0.17	0.30	0.19	0.66*	0.17
ITERS-R/ECERS-R gain score	0.19*	0.09	0.18*	0.08	0.02	0.10	−0.11	0.09	−0.01	0.10	0.00	0.09
SR pretest	0.26*	0.08	–	–	–	–	–	–	–	–	–	–
RA pretest	–	–	0.38*	0.07	–	–	–	–	–	–	–	–
SL pretest	–	–	–	–	0.12	0.08	–	–	–	–	–	–
VC pretest	–	–	–	–	–	–	0.36*	0.07	–	–	–	–
DS pretest	–	–	–	–	–	–	–	–	0.17*	0.09	–	–
FPPI pretest	–	–	–	–	–	–	–	–	–	–	−0.06	0.08
*Random parameters*												
Center level	0.00	0.00	0.00	0.00	0.00	0.00	0.00	0.00	0.05	0.13	0.03	0.11
Group level	0.21	0.10	0.20	0.08	0.15	0.11	0.21	0.09	0.27	0.17	0.12	0.14
Caregiver level	0.62	0.10	0.48	0.08	0.81	0.14	0.55	0.09	0.64	0.11	0.74	0.12
*Deviance*	363.76		332.93		387.24		351.19		378.90		374.35	

*SR* sensitive responsiveness, *RA* respect for autonomy, *SL* structuring and limit setting, *VC* verbal communication, *DS* developmental stimulation, *FPPI* fostering positive peer interactions

* *p* < .05

## Discussion

This study examined the effects of the CIP training, a 5-week video feedback training for caregivers in child care centers aimed at strengthening their interactive skills as measured with the CIP scales. Results at posttest indicate that the training had a positive effect on all six caregiver interactive skills, with a medium to large effect size for sensitive responsiveness, respect for autonomy, verbal communication, developmental stimulation, and fostering positive peer interactions, and with a small to medium effect size for structuring and limit setting. Three months after the posttest, at follow-up, caregivers in the experimental group still scored significantly higher than caregivers in the control group on sensitive responsiveness, respect for autonomy, verbal communication, and fostering positive peer interactions, with medium to large effect sizes. Altogether, this study shows that the quality of caregiver–child interactions can be improved for all six important caregiver skills, with a relatively short training program.

The effect sizes reflecting the improvement in caregiver skills at posttest ranged between medium and large. Compared to the aggregated effect sizes at posttest in the two meta-analyses described in the introduction (Fukkink and Lont 2007; Werner et al. 2016), effects of the current CIP training are somewhat larger, indicating that the effects of the CIP training on caregiver interactive skills are above-average immediately after the training. Moreover, posttest effect sizes for previous training programs that were merely aimed at sensitive responsiveness yielded a medium effect size (Werner et al. 2016), while the present CIP training reported nearly a large effect size for sensitive responsiveness. Also, effect sizes for verbal communication and fostering positive peer interaction in the present study are notably larger than the effect sizes reported in previous studies. Neither of the meta-analyses reported effect sizes at follow-up and, therefore, no direct comparison can be made for retention.

Although the CIP training improved both the more ‘basic’ caregiver skills and the ‘educational’ skills (i.e., verbal communication, developmental stimulation and fostering positive peer interactions), the improvement of the latter skills is especially relevant because these skills are of particular concern in The Netherlands. The pretest scores in the present study (Table 2) and the results of an earlier study in a large Dutch representative sample of caregivers (De Kruif et al. 2009; Helmerhorst et al. 2015) showed low to moderate average scores for verbal stimulation and very low to low average scores for developmental stimulation and fostering positive peer interactions. The improvement of caregivers’ educational skills may, in turn, positively affect children’s language, cognitive, and social development, as suggested by the results of earlier research summarized in the theoretical underpinning of the CIP scales in the introduction of this paper. Furthermore, the improved caregiver educational skills may also contribute to the reduction of negative peer interactions. Earlier research in Dutch child care centers has shown that negative peer interactions occur frequently during the child care day and that such negative interactions are stable over time and predict children’s aggression in later years (Gevers Deynoot-Schaub and Riksen-Walraven 2006). The relatively high proportion of negative peer interactions in child care centers could be explained by children’s limited verbal and social abilities. Therefore, the improvement of caregivers’ verbal communication with the children and an improved ability to foster positive peer interactions might also indirectly—through improvement of children’s verbal abilities and social competence—contribute to the reduction of negative peer interactions and aggression in the child care group. To our knowledge, this is first study showing that it is possible to improve caregivers’ ability in fostering positive peer interactions as a separate caregiver skill. This highly relevant aspect of the CIP training sets it apart from well-known existing training programs such as MyTeachingPartner.

Despite the significant improvement in the educational skills after training, with medium to large effect sizes, the average posttest scores on these scales were still in the low to moderate range, leaving much room for further improvement. The low level of educational skills in Dutch caregivers may be due to the curriculum of the regular vocational education in general social-pedagogic work that was completed by the vast majority of caregivers in our sample. This education prepares them to provide care for a broad age group (from children to elderly) and has a strong emphasis on care rather than on stimulating young children’s socio-emotional and cognitive development. So, especially with regard to the educational skills, the CIP training may have had no strong foundation to build on, which may explain why the posttest-levels—although higher than the pretest levels—were still low. Evidently, a 5-week training with only limited attention to the educational skills is not enough to produce a large and enduring improvement.

The training results for developmental stimulation and fostering positive peer interactions might be improved by adding extra training sessions, given that the initial level for these skills was relatively low. In the present training, these two caregiver skills are addressed in the final sessions of the training and might therefore get less attention and less opportunity than the earlier addressed skills to be strengthened and integrated in the caregivers’ everyday behavioral repertoire.

To prevent that the improvement in caregiver skills decreases after the training (a beginning of which is already visible in a decreasing effect sizes from posttest to follow-up for four of the six caregiver skills), we also recommend a monitoring system after the training. This could be realized by using a system comparable with MyTeachingPartner of the CLASS (see Pianta et al. 2008b). In this training, caregivers upload their own videos online and receive online feedback on their interactive skills complemented with concrete actions points by a trainer. Another, possibly more efficient long-term solution to ensure monitoring would be to provide an in-company training for staff members of the child care organizations to assess and monitor their caregivers’ interactive skills. This would require a *train the trainer* program, which we are currently developing. The first phase of the train the trainer program is devoted to training the staff members in reliably observing caregivers’ interactive skills with a simplified version of the CIP scales, with high/medium/low ratings instead of a 7-point scale. In a next step, we train the staff members in providing video feedback to the caregivers. Future research should make clear whether the abovementioned adaptations contribute to the effectiveness of the CIP training, also in the long run.

### Limitations and Future Directions

Some limitations of this study should be noticed. First of all, the present CIP training for caregivers was conducted parallel to a consultancy program for directors of the same centers. Although the consultancy program was not directed at improving caregiver interactive skills and although we controlled for possible effects of the consultancy program, possible confounding cannot be completely ruled out as an explanation for the effects of the CIP training. Further research examining the effects of an independently conducted CIP training may clarify this question.

Second, due to the fact that some child care centers participated with both groups in the experimental and in the control condition, possible diffusion of treatment could not be ruled out and is a limitation of the study. However, the training design was highly individualized with video examples of caregiver’s own functioning and personalized feedback; therefore, diffusion of treatment seems unlikely. Nevertheless, we acknowledge this as an important limitation.

A third limitation is that the results of this study cannot be generalized to the general population of Dutch child care centers, because our sample was not based on random selection. Also, the positive results do not automatically imply that this training is also effective in other countries. The effectiveness of the training may depend, for example, on caregiver education, which shows considerable variation across countries (Oberhuemer et al. 2010). It should also be kept in mind that the CIP scales were initially developed for use in child care centers in the Netherlands and therefore reflect Dutch child care values. As described in an earlier study (De Kruif et al. 2007; Helmerhorst et al. 2014) the choice of the six caregiver skills included in the CIP was also based on the results of a survey among different groups of stakeholders in child care, which showed that Dutch parents, caregivers, center directors, and external experts recognized these skills as important child care quality indicators. Although the caregiver skills included in the CIP scales are generally recognized as important by researchers and are also included in measures developed in other countries (e.g., the ORCE, see NICHD ECCRN 1996; and the CLASS, see La Paro et al. 2004), it cannot be automatically assumed that these skills are also seen as key aspects of pedagogical quality in other countries.

A final limitation is that not all caregivers per care group were trained. For practical reasons, we only trained the caregivers that were present during the day of the pretest observations. Because most caregivers work part-time, it is very common in the Netherlands that children are cared for by more than three caregivers during the week. Prior research has shown that caregiver behavior is for the most part determined by individual caregiver characteristics, which suggests that children may experience large differences in the quality of interactions with all the different caregivers in the care group (Helmerhorst et al. 2014). Therefore, to get a complete picture of children’s everyday experiences in the care group, it would be better to observe all caregivers of the care group. To improve the quality of the children’s everyday experiences in the care group, it would be beneficial to train all caregivers to strengthen their interactive skills. For future research, we therefore recommend to include all caregivers of the care group in a CIP training. Taking this a step further, it would also be interesting to not only examine the effects of the CIP training on the caregivers’ interactive skills, but to also examine its effects on the development and functioning of the children in their care group. This would also make it possible to examine whether including more caregivers per care group in the CIP training will indeed increase the effect of the training on the children. Furthermore, training all caregivers of a care group may not only positively affect the children, but it could also improve the retention of training effects and fulfillment of the training goals, as caregivers may reinforce each other’s efforts and continue to model and learn from each other.

Given that the present study sample only included female caregivers, possible effects of caregiver gender could not be examined. This is an interesting topic for future research. As far as we know, only two studies have compared the quality of caregiver–child interactions in male versus female caregivers and these studies have yielded mixed results. One study has found the quality of caregiver–child interactions to be more positive an less punitive with male than with female caregivers (Aigner et al. 2013), but another study showed no differences between male and female caregivers in the quality of caregiver–child interactions (Brandes et al. 2015).

Finally, the present study was carried out in mixed age groups with children across the whole age range between 0 and 4 years, because these groups are predominant in Dutch child care centers. Future research is needed to examine whether the effects of the CIP training are generalizable to caregivers in same-age groups.

### Implications for Policy and Practice

The results of the present study demonstrate that it is possible to strengthen the theoretically most important skills of professional caregivers in interacting with 0- to 4-year old children through a relatively short training program. This is a very promising finding, given that these caregiver interactive skills constitute the core of high-quality child care for young children and have been shown in earlier research to contribute to children’s wellbeing and development. The finding that these key interactive caregiver skills can indeed be strengthened by training and that training gains may even be retained for a longer period, makes it worthwhile to invest in this type of training.

The present training could be made available to the child care field to booster the interactive skills of caregivers in child care centers. The present set-up of the training, however, may be too financially challenging in case of large-scale implementation. Therefore, we are currently working on an adjustment of the training to make implementation in practice more feasible. Although prior research and the present study suggest that training caregivers individually is an important element in effective programs, it seems worthwhile to examine the possibility of training all caregivers of the care group together in focus groups. The advantage could be that caregivers learn from each other from the beginning instead of only during the last session, which may in turn lead to better retention of the gains made during training, because caregivers could monitor each other afterwards. Pilot studies are needed to examine whether the program could also be effectively used when all caregivers of the care group are trained together.

Another possibility to utilize the present training to improve caregiver interactive skills and thereby the quality of early child care is to incorporate the training in the regular education of caregivers. The current regular vocational education for caregivers in early child care does not educate students adequately in how to interact with young children in child care centers. Therefore, a straightforward next step would be to adapt the CIP training for implementation in the regular caregiver education, which requires the development of a *train the trainer* program for teacher educators in caregiver education. We are currently developing an adapted version of the training and will examine the effectiveness of its implementation in caregiver education in future research.

The most powerful way to attain and retain improvement of the quality of caregiver–child interactions in early child care centers would be to combine the two abovementioned applications of the caregiver interactive skills training. This means that the future approach for enhancing the quality of care and education for young children should be twofold: training caregivers who already work in the field *and* investing in the interactive skills of future caregivers by implementing the CIP training in the curriculum of caregiver education.
